# Recent Developments of Gramine: Chemistry and Biological Activity

**DOI:** 10.3390/molecules28155695

**Published:** 2023-07-27

**Authors:** Jiaoyue Zhang, Qitao Jia, Na Li, Liqiang Gu, Wenjia Dan, Jiangkun Dai

**Affiliations:** 1 School of Life Science and Technology, Weifang Medical University, Weifang 261053, China; zjiaoyue9637@163.com (J.Z.); qjia@wfmc.edu.cn (Q.J.); 2 Instrumental Analysis Center, Xi’an Jiaotong University, Xi’an 710049, China; lina2021@xjtu.edu.cn; 3 School of Materials Science and Engineering, Shandong University of Technology, Zibo 255000, China; gulq123@163.com

**Keywords:** gramine, extraction, synthesis, biological activity, mechanism

## Abstract

The natural alkaloid gramine has attracted significant attention in both academic and industrial circles because of its potential and diverse biological activities, including antiviral, antibacterial, antifungal, anti-inflammatory and antitumor activities; application in therapy for Alzheimer’s disease; serotonin-receptor-related activity; insecticidal activity; and application as an algicide. In this review, we focus on the research advances that have been made for gramine-based molecules since their discovery, providing key information on their extraction and separation, chemical synthesis and diverse biological activities. Data regarding their mechanisms of action are also presented. This comprehensive and critical review will serve as a guide for developing more drug candidates based on gramine skeletons.

## 1. Introduction

**Figure 1:**
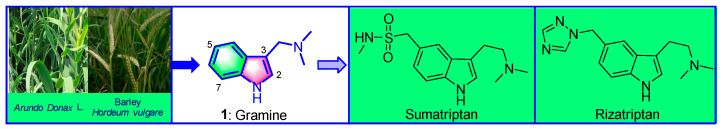
Structures of gramine, sumatriptan, and rizatriptan.

Gramine **1**, also known as *N*,*N*-dimethyl-1*H*-indole-3-methylamine (Figure 1), is an indole alkaloid initially isolated from *Arundo donax* L. and usually plays a defensive role in plants against herbivores [1]. Recently, this alkaloid was also isolated from various raw plants, particularly barley, and it could act as a precursor for the biosynthesis of tryptophan and play a vital role in amino acid metabolism [2]. Gramine has attracted much attention due to its diverse antiviral, antibacterial, anti-inflammatory, antitumor and insecticidal activities [3]. In traditional Chinese medicine, it is also used to control toothache, bad urination, and heart disease [4]. Interestingly, some molecules similar in structure to gramine, such as sumatriptan and rizatriptan, have been approved for clinical trials or for use in clinical practice [5].

The chemical synthesis of gramine was reviewed by Semenov et al. in 2004 [6]. However, since then, the following features have not yet been reviewed, or they have only been reviewed in a very fragmented fashion: (i) the extraction and separation of gramine; (ii) the chemical synthesis of gramine and its derivatives; and (iii) the diverse biological activities. This review aims to provide a detailed account of the chemistry and biological activities of gramine-based molecules that have been explored since their discovery. For the sake of convenience, their extraction and separation are first summarized. Then, the chemical synthesis of gramine is presented according to the key reaction. In the following section, its function and biological activities are presented, including its antiviral, antibacterial, antifungal, anti-inflammatory and antitumor activities; application in therapy for Alzheimer’s disease; serotonin-receptor-related activity; insecticidal activity; and application as an algicide. Finally, we offer a conclusion and suggest future research directions on the topic of gramine based on our expertise in this field.

## 2. Extraction and Separation

*Arundo donax* L., a perennial cane, grows in damp soils, and it is widely distributed in mild–temperate subtropical and tropical regions, such as the western Pacific and Mediterranean [7]. In 1935, Orechoff et al. first discovered gramine in *A. donax* L. and named it donaxin [1]. Interestingly, in 1959, gramine was first discovered in *Acer saccharinum*, with 1.1 g of prismatic crystals obtained from 3.75 kg of dried maple leaves, which are widely distributed in the United States and southeastern Canada [8]. Then, in 1976, Anderson et al. extracted 85 mg of gramine from 640 g of seeds of *Lupinus hartwegii* (six weeks old) grown in vermiculite [9]. In 1985, Zúǹiga et al. reported gramine in 34 barley cultivars, ranging in amounts from 0 to 48 mmol/kg [10]. Barley is a major cereal grain that is widely planted in temperate climates. Specifically, the content of gramine in barley leaves decreased with age. Moreover, its concentration in dry barley cultivars could reach up to 8 mg/g [11]. In 2020, an ultrasonic method was used to extract gramine [4]. The extraction rate of gramine from *A. donax* L. was 1% under the following conditions: ultrasonic power: 600 W, time: 50 min, temperature: 50 °C, liquid–material ratio: 40 mL/g, and pH value: 5.

## 3. Chemical Synthesis

Gramine and its derivatives have been synthesized via different methods by the scientific community due to their attractive function and biological activities. With readability in mind, we have summarized these classic and efficient synthetic methods according to their key reaction type.

### 3.1. Mannich Reaction

#### 3.1.1. Acetic-Acid-Catalyzed Reaction

**Scheme 1:**
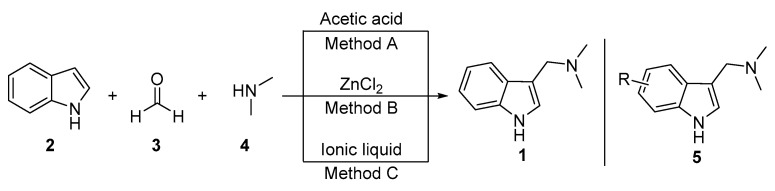
The synthetic methods based on the Mannich reaction.

The Mannich reaction (Scheme 1) is widely employed in the synthesis of gramine and its derivatives, with indole, formaldehyde, and dimethylamine used as the raw materials. In 2004, Xu et al. carried out the synthesis of gramine **1** using acetic acid as the catalyst, with the total yield optimized to 95.6% [12]. In 2020, Zhang et al. showed the synthesis of gramine **1** and its analogue **5**, which were substituted by methyl, nitro, and methoxy groups, with yields ranging from 70.5% to 95% [13]. In 2014, microwave-assisted technology was introduced to the acetic-acid-catalyzed Mannich reaction, with a high yield of 98.1% [14]. Notably, the reaction time is only 5 min.

**Scheme 2:**
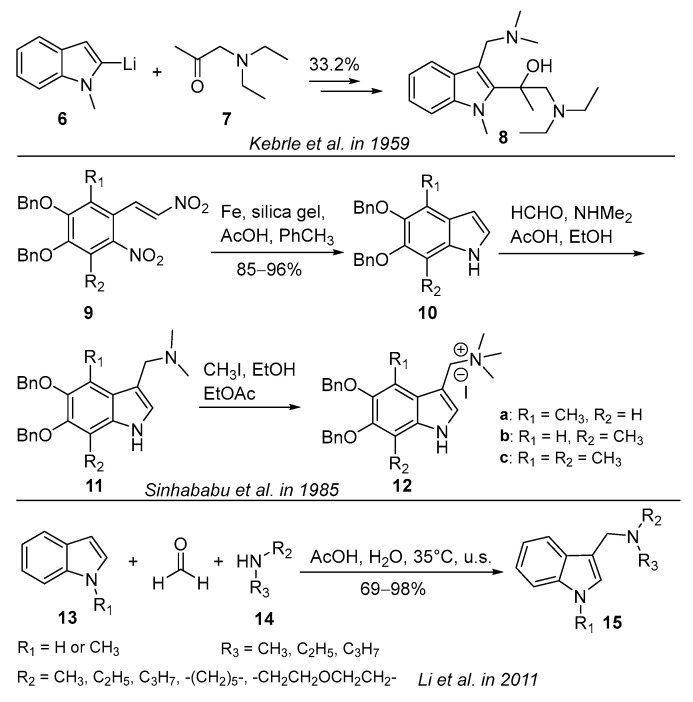
Synthesis of gramine derivatives **8**, **11**, **12** and **15** [15,16,17].

In addition, a great deal of gramine derivatives were also prepared using an acetic-acid-catalyzed Mannich reaction. In 1959, compound **8** was synthesized as an intermediate to prepare *γ*-carboline analogue with 2-lithium-1-methyl-indole **6** as raw reagents (Scheme 2) [15]. In 1985, some gramine-based quaternary ammonium salts **12a**–**12c** were obtained by Sinhababu et al. [16]. Notably, the reductive cyclization of compound **9** to compound **10** using an Fe/AcOH system provided poor yields. When silica gel was added to toluene, compound **10** could be obtained with yields of 85–96%. In 2011, compound **15** was prepared in a Mannich reaction under ultrasound irradiation, with yields ranging from 69% to 98%, which was milder and more efficient than the traditional stirring method [17].

**Scheme 3:**
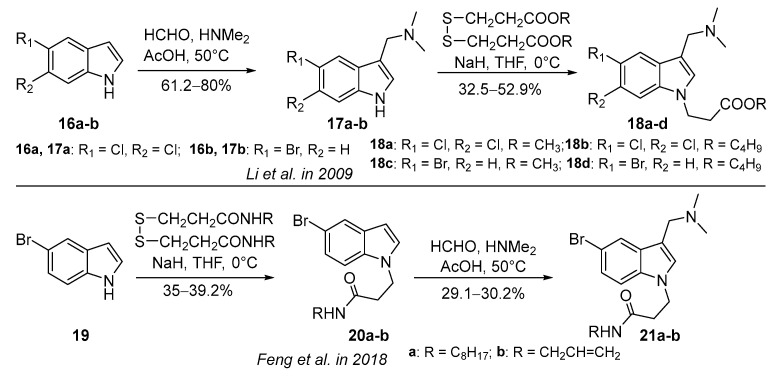
Synthesis of gramine derivatives **17**, **18** and **21** [18,19].

In 2009, some gramine analogues, such as compounds **17** and **18**, containing ester functional groups were prepared and evaluated for their algal inhibition activity (Scheme 3) [18]. Notably, 3,3′-dithiobispropionate was used to provide the ester functional groups in the presence of sodium hydride. In 2018, compound **21** was synthesized using a similar method, and its antibacterial and antifouling activities were studied [19].

#### 3.1.2. Zinc-Chloride-Catalyzed Reaction

**Scheme 4:**
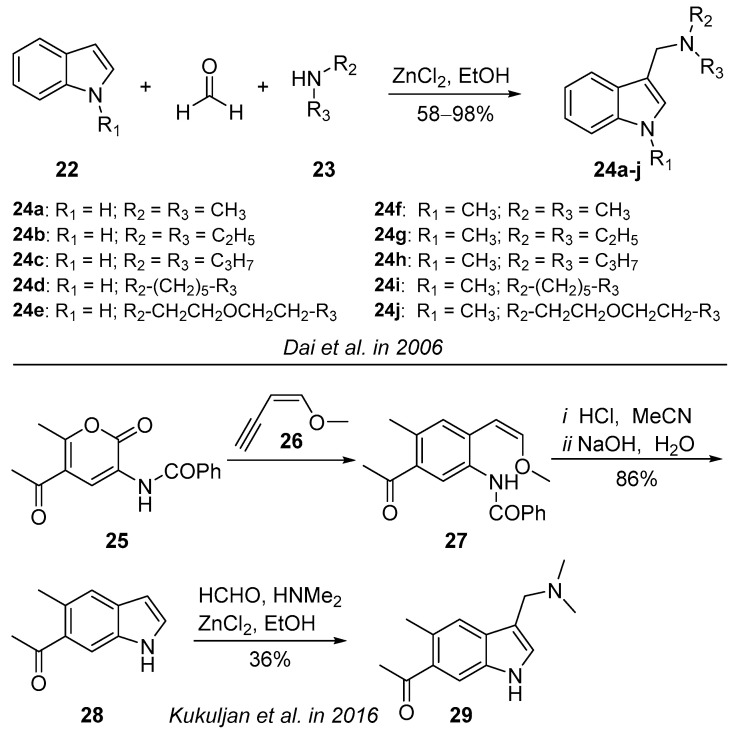
Synthesis of gramine derivatives **24** and **29** [20,22].

Zinc chloride has been widely used in organic reactions, including the Mannich reaction. In 2006, a series of gramine derivatives **24** were obtained at room temperature using ZnCl_2_ as a catalyst in EtOH (Scheme 4) [20]. In 2015, an immobilized montmorillonite (MMT)/ZnCl_2_ system was used to synthesize gramine **1** via a microwave-assisted method. The yield reached 93.8% with a reaction time of 5 min, and the yield was still 60% after being recycled three times [21]. In 2016, compound **27** was prepared using the Diels–Alder cycloaddition reaction, with compound **26** as the dienophile. Then, the substituted indole **28** was obtained via acid-catalyzed cyclization and concomitant deprotection, which further derivatized to the gramine analogue **29** via the Mannich reaction [22].

#### 3.1.3. Ionic-Liquid-Catalyzed Reaction

In 2015, acidic ionic liquid, which is more suitable and reusable than classical acid catalysts, was used as a catalyst to prepare gramine **1** in a Mannich reaction with a yield of 81.6%. Notably, 1-butyl-3-methyl imidazole hydrogen sulfate can be continuously used as a catalyst. After four uses, the yield remained relatively unchanged [23].

### 3.2. Lithiation Reaction

**Scheme 5:**
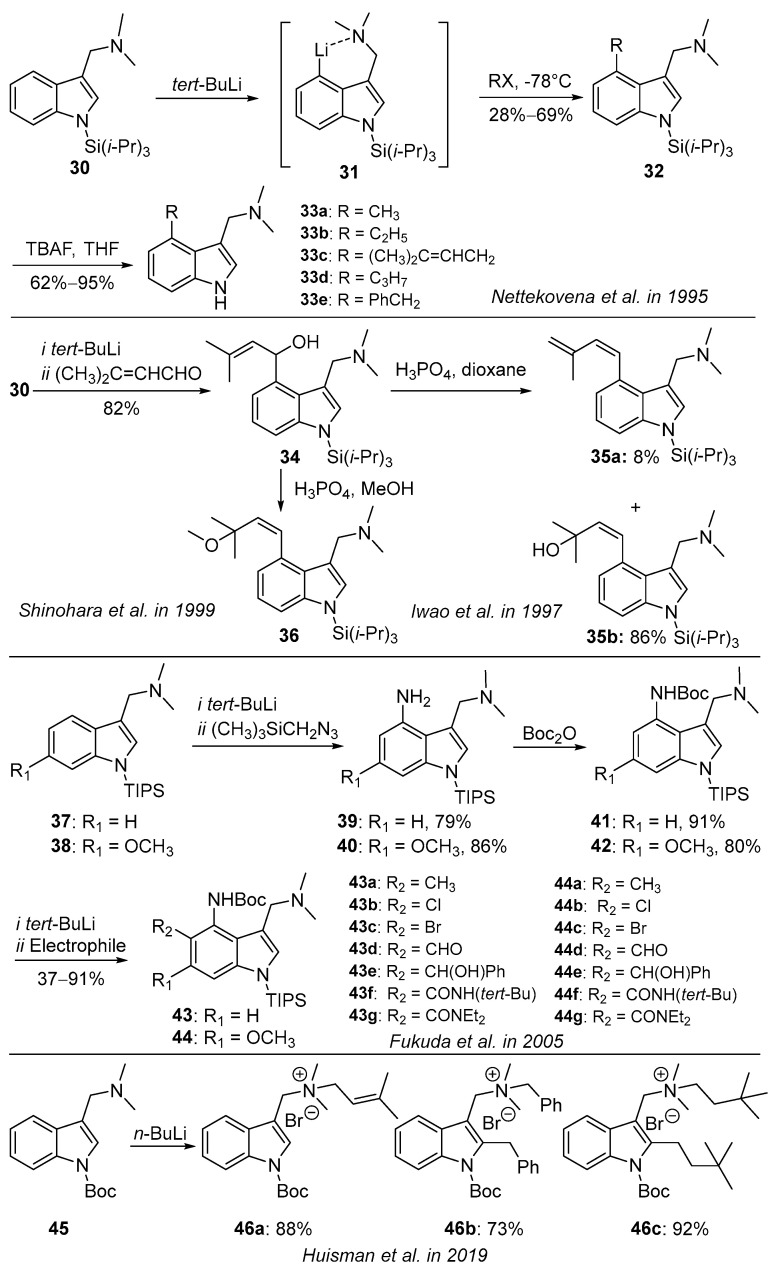
Synthesis of gramine derivatives **32**–**46** [24,25,27,28].

In 1995, the *N*-protected gramine **30** was selectively lithiated and yielded the organometallic intermediate **31**. Then, the 4-substituted gramine derivatives **32** and deprotected products **33** were obtained (Scheme 5) [24]. In 1997, some 4-substituted gramine derivatives **34**–**36** were synthesized via a lithiated reaction with compound **30** as the substrate [25]. In the same study, compound **36** was further synthesized based on compound **34**, with H_3_PO_4_/MeOH used as the reaction system [26]. In 2005, 4-amino-1-(triisopropylsilyl)gramines (**39** and **40**) were also synthesized via the directed lithiation of compounds **37** and **38**. Moreover, a variety of 5-functionalized compounds **43** and **44** were obtained using lithiated species and diverse electrophiles with yields of 37–91% [27]. In 2019, some gramine salts **46** were synthesized as intermediates for the synthesis of tryprostatin B by lithiation reaction [28].

### 3.3. Alkylation Reaction

**Scheme 6:**
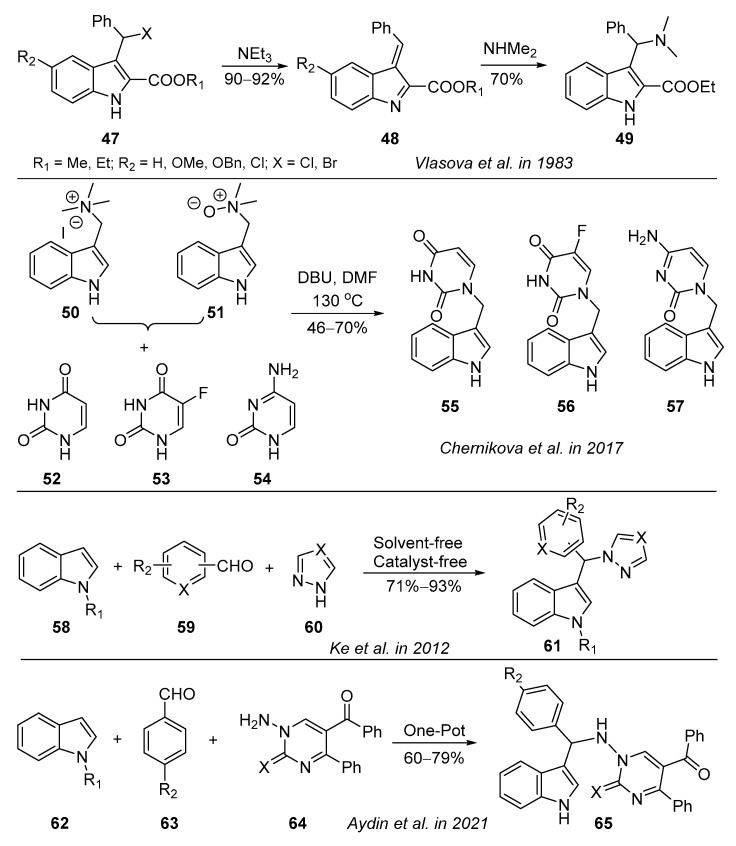
Synthesis of gramine derivatives **49**, **55**, **56**, **57**, **61** and **65** [29,30,31,32].

In 1983, some substituted gramines such as compound **49** were synthesized via the alkylation of amines (Scheme 6) [29]. In 2017, some gramine derivatives **56**–**57** that fused with nitrogen heterocyclic ring were synthesized via a reaction of compounds **52**–**54** with gramine *N*-oxide **51** or gramine methyl iodide **50** [30]. However, compounds **50** and **51** could convert to gramine **1** when the temperature was raised to 160 °C in the presence of compound **52**.

### 3.4. Three-Component Condensation Reaction

In 2012, some heterocycle-functional gramine analogues **61** were prepared via a convenient three-component reaction using a solvent- and catalyst-free method (Scheme 6) [31]. Inspired by Ke’s method, a series of gramine derivatives **65** fused with a pyrimidine ring were synthesized via a one-pot, three-component condensation reaction, providing an efficient, convenient, and green strategy [32].

### 3.5. Other Reactions

**Scheme 7:**
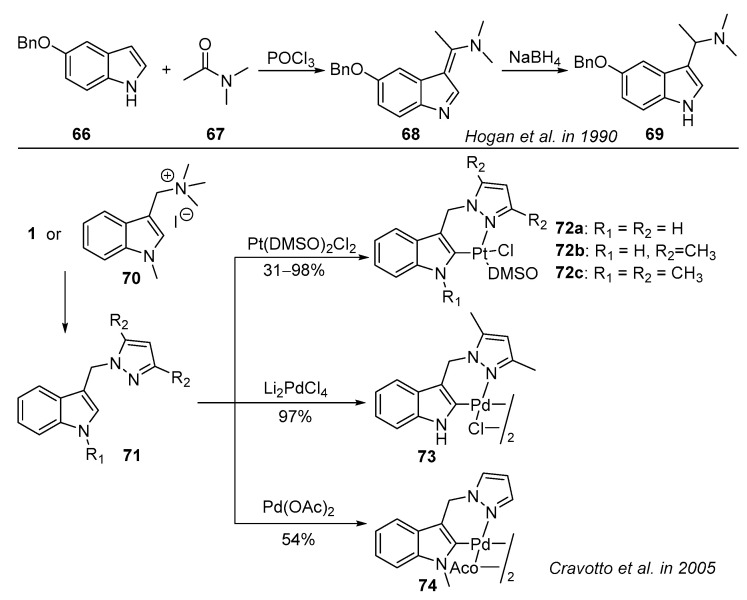
Synthesis of gramine derivatives **69**, **71**, **72**, **73** and **74** [33,34].

In 1990, *N*, *N*-dimethylacetamide was used to construct the gramine analogue **69** in the presence of POCl_3_ with a total yield of 33%. Notably, the intermediate **68** without purification was further reduced by NaBH_4_ (Scheme 7) [33]. In 2005, some Pt (II) and Pd (II) complexes **72**–**74** based on a gramine skeleton were synthesized and used as catalysts in a Heck reaction [34]. As shown in Scheme 7, gramine analogues **71** that fused with pyrazole and 3,5-dimethylpyrazole can be easily synthesized using gramine **1** or compound **70**. Then, the target compounds **72**–**74** can be obtained in the presence of Pt(DMSO)_2_Cl_2_, Li_2_PdCl_4_, and Pd(OAc)_2_, respectively.

## 4. Biological Activities

### 4.1. Antiviral Activity

**Figure 2:**
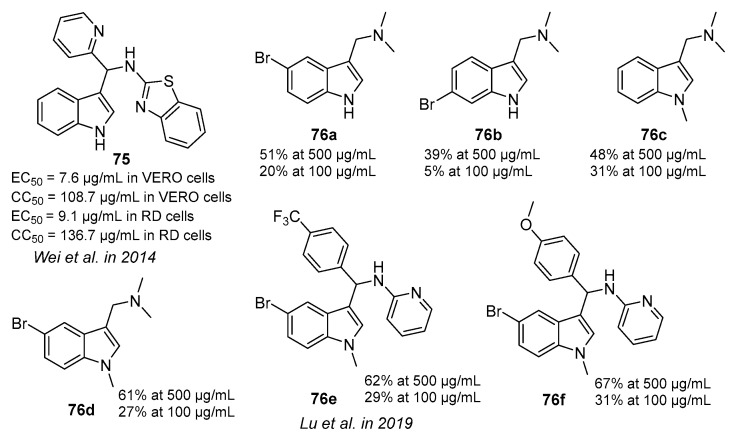
The structures and antiviral activity of compounds **75** and **76** [38,39].

Enterovirus 71, widely reported in the Asia–Pacific region, is a classical RNA virus, which could infect the hand, foot and mouth of a human [35]. The prevention of this disease thus far has mainly relied on public health alerts and management [36]. Moreover, no specific medication for enterovirus 71 infections has thus far been used in clinical practice. Usually, some broad-spectrum antiviral drugs, such as type I interferon, ribavirin, and pleconaril, are used for infection treatment of enterovirus 71 [37]. In 2014, some gramine derivatives synthesized by Wei et al. exhibited potential inhibition activity on enterovirus-71-induced cytopathic effects (Figure 2). The EC_50_ values for compound **75** against enterovirus 71 in African green monkey kidney cells (Vero) and rhabdomyosarcoma cells (RD) were 7.6 and 9.1 μg/mL, respectively, which were more potent than ribavirin (44.6 and 32.1 μg/mL). Interestingly, it exhibited high selectivity with CC_50_ values greater than 100 μg/mL. The study of the antiviral mechanism revealed that compound **75** acted in the early stage of the enterovirus 71 lifecycle; this compound could significantly intervene in protein synthesis, RNA replication, and enterovirus-71-induced apoptosis in RD cells [38].

Tobacco mosaic virus (TMV), first discovered in tobacco, can infect as many as 400 different crops and lead to enormous economic losses [40]. The commercial pesticide, ningnanmycin, is currently the most competitive antiviral product; however, it only exhibits 50–60% control at a concentration of 500 μg/mL [40]. As shown in Figure 2, compared with the inhibition rate of gramine **1** (23%), bromination analogues (**76a** and **76b**) exhibit higher antiviral activities with inhibition rates of 51% and 39% at 500 μg/mL, respectively. Methylation at the *N*^1^ position could also improve anti-TMV activity, such as in compound **76c**. Interestingly, the simultaneous substitution of bromination at the *C*^6^ position and methylation at *N*^1^ position caused a synergistic effect, such as in compound **76d,** with an inhibition rate of 61% at 500 μg/mL. In addition, the introduction of aryl substituents at the methylene of the flexible chain could further enhance anti-TMV activity, such as in compounds **76e** (62%) and **76f** (67%). A study of the anti-TMV mechanism revealed that compound **76** could block the assembly of TMV via cross-linking the TMV capsid protein [39]. Influenza A virus (IAV) is a kind of respiratory pathogen with high infectivity and pathogenicity, belonging to the family of *Orthomyxoviridae*. In 2021, Zhao et al. studied the inhibition activity of gramine **1** in IAV. Regrettably, no anti-IAV activity was found [41].

### 4.2. Antibacterial Activity

In 2014, Yang et al. reported the antibacterial activity of gramine **1**, which could effectively inhibit the growth of *Escherichia coli* and *Staphylococcus aureus* with minimum inhibitory concentrations (MICs) of 16.92 and 6.26 μg/mL [42]. Moreover, it could also mildly inhibit the growth of methicillin-resistant *S. aureus* (MRSA) with an inhibition rate of 82% at 400 μg/mL [43]. In 2018, Feng et al. reported the antibacterial activity of gramine derivatives associated with the acylamino group [19]. Interestingly, compounds **21a** and **21b** exhibited moderate antibacterial activity against *S. aureus* with an MIC of 30 μg/mL. The rhizosphere microbiota can reflect the growth and development status of plants, which is valuable for sustainable agriculture. In 2021, Maver et al. found that gramine could regulate the prokaryotic communities of barley rhizosphere microbiota [44]. In the same year, these results were also validated by Schütz et al. [45]. They found that gramine **1** could promote the proliferation of beneficial strains, such as *Novosphingobium* and *Massilia* ASVs.

### 4.3. Antifungal Activity

**Figure 3:**
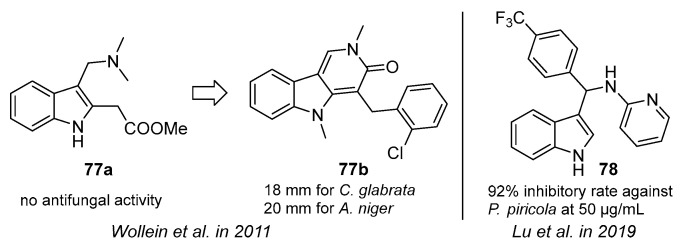
The structures and antifungal activity of compounds **77**–**78** [39,47].

In 2001, Matsuo et al. found that the content of gramine **1** significantly increased in the leaves of barley seedlings after being infected by *Blumeria graminis*, which indicated its potential antifungal activity [2]. In 2011, Schreiber et al. reported that gramine **1** could effectively decrease the severity of *Fusarium graminearum* infection in wheat [46]. In 2011, Wollein et al. studied the antifungal activity of gramine derivative **77a** against *Candida glabris* and *Aspergillus Niger* (Figure 3). Unfortunately, no inhibition activity was found. However, its cyclization product **77b** exhibited antifungal activity against *C. glabris* and *A. Niger* with the diameters of inhibitory zones being 11 and 10 mm, respectively [47]. In 2019, Lu et al. discovered that compounds **76e** and **78** exhibited more than a 90% inhibitory effect in vitro against the plant pathogen *Phytophthora piricola* at 50 μg/mL via the mycelial growth method [39].

### 4.4. Anti-Inflammatory Activity

Inflammation is loosely defined as a response to invading pathogens or endogenous signals, which plays a vital role in many diseases, especially in some chronic diseases [48]. 5-Lipoxygenase (LOX) enzymes, which are involved in leukotriene biosynthesis with arachidonic acid, could mediate inflammatory reactions [49]. The inhibition of the inflammatory factor nitric oxide (NO) has also been considered to be an anti-inflammatory strategy [50]. In 2017, Magalhães et al. reported that gramine **1** can inhibit the activity of LOX, with an IC_25_ value of 119 μg/mL. Moreover, it can effectively scavenge 34% of nitric oxide radicals at 1 mg/mL [51]. It has been suggested that the nuclear translocation of nuclear factor kappa B (NF-KB) and signal transducer and activator of transcription 3 (STAT 3) could transcribe inflammatory genes [52]. The activation of the tyrosine kinase phosphorylation of the epidermal growth factor receptor (EGFR) may initiate the Janus Kinases (JAKs) and phosphoinositide 3-kinase (PI3K)/protein kinase B (Akt)/mammalian target of rapamycin (mTOR) pathways [53]. In 2018, Ramu et al. reported that gramine **1** exhibited an anti-inflammatory activity via the intervention of EGFR/PI3K/Akt/mTOR/IKK/NF-κB and JAK/STAT3 signaling pathways [54]. In 2021, Lu et al. discovered that gramine **1** could inhibit the release of pro-inflammatory mediators, including interleukin-1β (IL-1β), IL-6, tumor necrosis factor (TNF)-α, and NO secreted from lipopolysaccharide (LPS)-induced microglia. Moreover, it was found to reduce the expressions of inducible nitric oxide synthase (iNOS) and cyclooxygenase-2 (COX-2). In vivo behavioral and histological experiments indicated that gramine **1** could alleviate microglia activation and promote motor functional recovery via the NF-κB pathway [55].

### 4.5. Antitumor Activity

**Figure 4:**
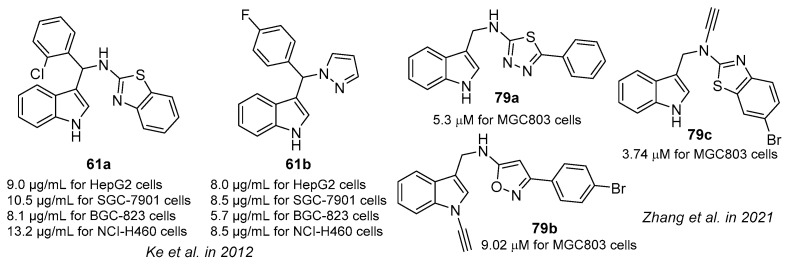
The structures and antitumor activities of compounds **61** and **79** [31,60].

Oral squamous cell carcinoma (OSCC), as one of the most lethal tumors, is one of the top six human malignancies [56]. 7,12 Dimethylbenz[a]anthracene (DMBA) induced OSCC in a hamster buccal pouch (HBP), which is widely used as an animal model [57]. In 2014, Kumar et al. reported that gramine **1** exhibited a potential chemopreventive effect on DMBA-induced HBP, which might be attributed to its anti-lipid peroxidative, antioxidant potential, and retrieval effects as well as detoxifying potential [58]. In 2017, they found that gramine **1** decreased angiogenesis and induced apoptosis in HBP by regulating transforming growth factor (TGF)-β signals [59]. In 2018, they further proved that gramine **1** could activate the operation of key cancer suppressor proteins p21, p53 as well as Gsk-3β, which explained the anti-proliferation effects [54]. In 2012, Ke et al. reported the anti-proliferation activity of compound **61** against human gastric cancer, human lung cancer, and human hepatocellular liver cancer cell lines (Figure 4). Among these, compound **61b**, with an IC_50_ value of 5.7 μg/mL, could inhibit the growth of BGC-823 human gastric cells [31].

In 2021, Zhang et al. also reported the anti-gastric cancer activity of a series of gramine-based hybrid molecules, including **79**. Compound **79c** exhibited good activity against MGC803 human gastric cancer cells (IC_50_ = 3.74 μM). A study of its mechanism revealed that compound **79c** could cause cell cycle arrest, induce mitochondria-mediated apoptosis, and inhibit metastasis [60]. In 2023, gramine-loaded, polyvinyl-alcohol-coated iron oxide nanoparticles were prepared by Alnaim et al. and exhibited a good anti-proliferation activity against HCT-116 human colon cancer cells, with an IC_50_ value of 25 μg/mL. The study of its mechanism indicated that these nanoparticles could destroy the equilibrium of redox pathways and cause cell apoptosis [61].

### 4.6. Alzheimer’s Disease Therapy

**Figure 5:**
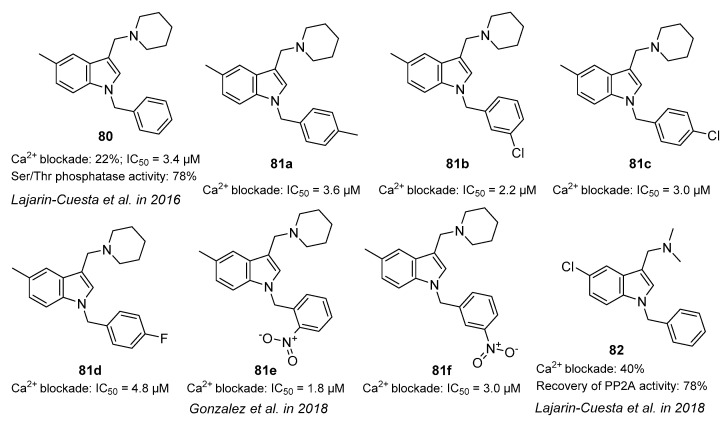
The structures and AD-related therapeutic activities of compounds **80**–**82** [66,67,68].

Alzheimer’s disease (AD), as a neurodegenerative disease, is an enormous burden on society [62]. The physiological characteristics of AD, including amyloid β peptide (Aβ) and neurofibrillary tangles (NFTs) caused by τ protein aggregation, are important therapeutic targets [63]. The increasing amount of phosphorylated τ protein causes it to self-aggregate into NFT, depending on the activity of kinase and phosphatase. Specifically, Ser/Thr phosphatase plays an important role in the dephosphorylation of the τ protein [64]. In addition, the reduction in cytosolic Ca^2+^ in neurons can block the development of AD, such as in the successful commercialization of memantine [65]. In 2016, Lajarín-Cuesta et al. reported that compound **80** could reduce the entry of Ca^2+^ via voltage-gated calcium channels (VGCC) and maintain the action of Ser/Thr phosphatase 2A (PP2A), which decreased τ hyperphosphorylation (Figure 5) [66]. In 2018, their group reported the activity of *N*-benzylated gramines **81** that dissipate the overload of neuronal Ca^2+^ [67]. The introduction of a nitro group in benzyl could greatly improve the blocking effect of VGCC with an IC_50_ of 1.8 μM for compound **81e**. Moreover, Lajarín-Cuesta et al. synthesized a gramine derivative **82**, which could restore 78% of the PP2A activity and blockade 40% of VGCC [68]. Previous research indicated that neuroinflammation was a therapeutic target in neurodegenerative diseases, such as AD [48]. The activation of NF-κB in neurons will promote their survival. In 2021, Yang et al. reported that gramine **1** prevented the apoptosis of PC12 cells, inhibited neuroinflammation via the NF-κB signaling pathway, and ultimately promoted the treatment of associated central nervous diseases such as AD [55]. In 2023, Jadhav et al. found that gramine **1** could remarkably restore memory in an amnesia model induced by scopolamine, indicating its potential in therapy for AD [69].

### 4.7. Serotonin Receptor-Related Activity

**Figure 6:**
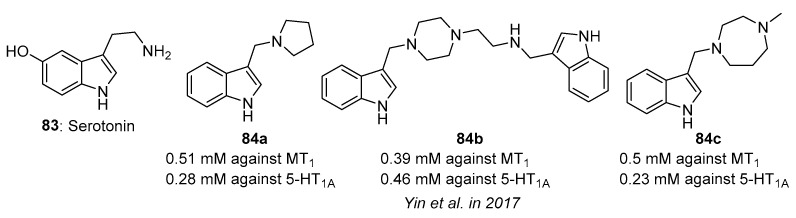
The structures and serotonin receptor-related activity of compound **84** [72].

Serotonin (5-HT, **83**), with seven known subtypes of receptors, is an important neurotransmitter and regulatory molecule that can regulate various physiological functions, such as appetite, sleep, mentation, emotions, etc. (Figure 6) [70,71]. Notably, different agonists and antagonists can be used in different receptor subtypes to treat different diseases [72]. Many studies have shown that gramine **1** is a 5-HT receptor antagonist due to its structural similarities. After being induced by dorsal swim interneurons (DSIs), 5-HT can mediate neurotransmission and neuromodulation. Gramine **1** can cause antagonistic effects by blocking the fast DSI–dorsal flexion neuron (DFN) excitatory postsynaptic potential (EPSP) and reducing the depolarization induced by 5-HT [73]. In addition, gramine **1** could decrease the 5-HT-induced metamorphosis of the gastropod mollusc, *Ilyanassa obsolete*, reducing it from 48% to 20% [74]. Litosch et al. reported that gramine **1** also antagonized the stimulation of cycloenzyme via 5-HT [75]. Renaud et al. found that gramine **1** could significantly delay the cleavage of sea urchins by stimulating calcium efflux from fertilized eggs and reducing the level of cAMP [76]. 5-HT can regulate the contraction time of the pharynx and increase the eating speed of *C. elegans* when food resources are sufficient. 5-HT can raise the rate of pharyngeal contraction by reducing the time needed for pharyngeal action. However, gramine **1** exhibited the opposite effect, which increases the duration of action potential [77]. In 2004, Froldi et al. found that gramine **1** as the antagonistic to 5-HT_2A_ receptor was an effective vasodilator [78].

It is well known that 5-HT is the direct precursor of melatonin (MT), which can enhance the activation of the 5-HT_1A_ receptor in the hypothalamus. There are three subtypes of MT receptor, MT_1_, MT_2_, and MT_3_, which are closely related to some mental disorders [79]. The EC_50_ values of gramine **1** on MT_1_ and 5-HT_1A_ were 1.36 and 0.47 mM, respectively [72]. Simultaneously, some derivatives **84a**–**84c** exhibited more potential activity as displayed in Figure 6, with the strongest EC_50_ value of 0.23 mM against 5-HT_1A_. Orchard et al. found that gramine **1** was also an antagonist of the octopusamine-2 receptor, which was used to block the increase in cyclic adenosine monophosphate mediated by octopamine [80].

### 4.8. Insecticidal Activity

**Figure 7:**
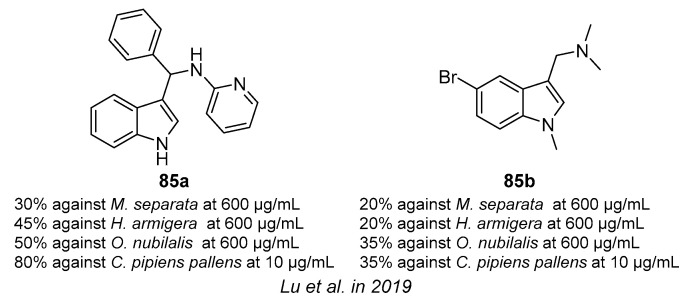
The structures and insecticidal activity of compound **85** [39].

Gramine **1**, as an important defensive toxin in plants, exhibits broad-spectrum insecticidal activity against herbivorous insects, including aphids, cotton bollworms, brown planthoppers and beetles [81]. In addition, gramine **1** also exhibits toxicity to *Daphnia magna* with an EC_50_ value of 6.03 μg/mL [82]. In 2019, Lu et al. explored the insecticidal activities of compounds **85a**–**85b** against *Helicoverpa armigera*, *Culex pipiens pallens*, *Ostrinia nubilalis* and *Mythimna separate* (Figure 7). Notably, the insecticidal rate of **85a** against *C. pipiens pallens* can reach 80% at the concentration of 10 μg/mL [39].

### 4.9. Algicides

The red tide and marine pollution caused by the large-scale flooding of algae seriously threatened the sustainable stability of the marine ecosystem [83]. *Prymnesium parvum*, a toxin-producing and harmful alga, is difficult to control. Interestingly, gramine **1** could significantly inhibit the growth of *P. parvum*, with IC_50_ values of 2.78 (3 days) and 1.83 μg/mL (9 days), respectively [84]. Specifically, its derivative 5,6-dichlorogramine **86** exhibited better activity, with IC_50_ values of 0.54 (3 days) and 0.22 μg/mL (9 days), respectively [84].

## 5. Conclusions and Perspectives

**Figure 8:**
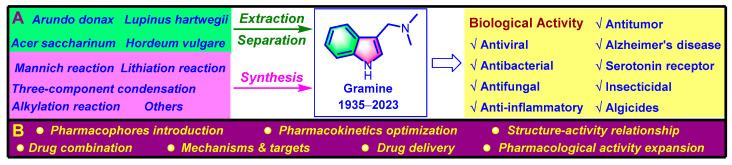
(**A**) Summary of the main findings in this review. (**B**) The perspectives for gramine-based medicinal chemistry research.

This review presents a wealth of information on gramine alkaloids, including their extraction, chemical synthesis, and diverse biological activities that have been discovered since their first isolation in 1935 (Figure 8A). Firstly, the source, distribution and extraction technology of gramine are briefly summarized. Then, their chemical synthesis methods are described according to their key reaction type. Clearly, gramine skeletons can be easily obtained. Modifications with various bioactive moieties provide many potential molecules. Indeed, some drugs similar in structure to gramine have been successfully marketed, such as sumatriptan and rizatriptan. Additionally, a literature search revealed that their biological activity mechanisms have also been carefully discussed. Of course, there remains much unreported information regarding pharmacological activity in gramine skeletons. For example, Xu et al. recently reported their therapeutic potential in pathological cardiac hypertrophy via an interaction with Runt-related transcription factor 1 [85]. Kozanecka-Okupnik et al. synthesized some triazole-bearing gramine analogues, which exhibited a protective activity against hemolysis induced by oxidative stress [86]. For medicinal chemists, gramine-based drugs will be used in the long term due to their simple structures and desirable activities.

New perspectives for gramine-based medicinal chemistry research can be divided into the following topics (Figure 8B): (a) introduction of key pharmacophores via the design of hybrid molecules; (b) structural optimization for defects such as poor pharmacokinetics and bioavailability; (c) elucidation of structure–activity relationships; (d) drug combination and drug resistance research; (e) in-depth exploration of diverse molecular mechanisms and targets, such as the application of multi-omics analysis; (f) efficient delivery forms of drug molecules based on gramine skeletons; and the (g) discovery and expansion of diverse pharmacological activity.

## Data Availability

Not applicable.

## References

[1] Orechoff A., Norkina S. (1935). Über die Alkaloide von *Arundo donax* L.. Ber. Dtsch. Chem. Ges..

[2] Matsuo H., Taniguchi K., Hiramoto T., Yamada T., Ichinose Y., Toyoda K., Takeda K., Shiraishi T. (2001). Gramine increase associated with rapid and transient systemic resistance in barley seedlings induced by mechanical and biological stresses. Plant Cell Physiol..

[3] Hanson A.D., Ditz K.M., Singletary G.W., Leland T.J. (1983). Gramine accumulation in leaves of barley grown under high-temperature stress. Plant Physiol..

[4] Li L., Yang P., Li X., Pan F., Yan H. (2020). Optimization of extracting process of gramine from *Arundo donax* by response surface method. Food Ind..

[5] Lines C.R., Visser W.H. (2001). Rizatriptan: Pharmacological differences from sumatriptan and clinical results. Curr. Med. Res. Opin..

[6] Semenov B.B., Granik V.G. (2004). Chemistry of N-(1H-indol-3-ylmethyl)-N,N-dimethylamine (gramine): A review. Pharm. Chem. J..

[7] Corno L., Pilu R., Adani F. (2014). *Arundo donax* L.: A non-food crop for bioenergy and bio-compound production. Biotechnol. Adv..

[8] Pachter I.J., Zacharias D.E., Ribeiro O. (1959). Indole alkaloids of *Acer saccharinum* (the silver maple), *Dictyoloma incanescens*, *Piptadenia colubrina*, and *Mimosa hostilis*. J. Org. Chem..

[9] Anderson J.N., Martin R.O. (1976). Aphylline, epiaphylline, 10,17-dioxosparteine, gramine, and other unexpected alkaloids from *Lupinus hartwegii*. J. Org. Chem..

[10] Zúǹiga G.E., Salgado M.S., Corcuera L.J. (1985). Role of an indole alkaloid in the resistance of barley seedlings to aphids. Phytochemistry.

[11] Zúñiga G.E., Corcuera L.J. (1986). Effect of gramine in the resistance of barley seedlings to the aphid *Rhopalosiphum padi*. Entomol. Exp. Appl..

[12] Xu Q., Wei P. (2004). Synthesis and application of gramine, a new plant-derived pesticide. Chin. J. Pestic. Sci..

[13] Zhang J., Meng G. (2020). Study on the synthesis of gramine and its derivatives. J. Dali Univ..

[14] Yin X., Wu W., Wu J., Qin S.H., Chen Z. (2014). Synthesis of gramine under microwave irradiation reaction. Agrochemicals.

[15] Kebrle J., Rossi A., Hoffmann K. (1959). Beiträge zur Chemie des Indols. Über eine neue Aufbaumethode von *γ*-Carbolinen. Helv. Chim. Acta.

[16] Sinhababu A.K., Ghosh A.K., Borchardt R.T. (1985). Molecular mechanism of action of 5,6-dihydroxytryptamine. Synthesis and biological evaluation of 4-methyl-, 7-methyl-, and 4,7-dimethyl-5,6-dihydroxytryptamines. J. Med. Chem..

[17] Li J.-T., Sun S.-F., Sun M.-X. (2011). Improved synthesis of 3-(dialkylaminomethyl)-indole in acetic acid aqueous solution under ultrasound irradiation. Ultrason. Sonochem..

[18] Li X., Yu L., Jiang X., Xia S., Zhao H. (2009). Synthesis, algal inhibition activities and QSAR studies of novel gramine compounds containing ester functional groups. Chin. J. Oceanol. Limn..

[19] Feng K., Li X., Yu L. (2018). Synthesis, antibacterial activity, and application in the antifouling marine coatings of novel acylamino compounds containing gramine groups. Prog. Org. Coat..

[20] Dai H.-G., Li J.-T., Li T.-S. (2006). Efficient and practical synthesis of Mannich bases related to gramine mediated by zinc chloride. Synth. Commun..

[21] Wu W., Wu X., Wu J., Yin X., Chen Z. (2015). Catalytic synthesis of gramine by immobilized montmorillonite under microwave irradiation. Chin. J. Synth. Chem..

[22] Kukuljan L., Kranjc K., Perdih F. (2016). Synthesis and structural evaluation of 5-methyl-6-acetyl substituted indole and gramine. Acta Chim. Slov..

[23] Zhang D., Peng X., Liu B., Guo X., Cao Q., Chao J. (2015). Synthesis of gramine using acidic ionic liquid as catalyst. CIESC J..

[24] Nettekoven M., Psiorz M., Waldmann H. (1995). Synthesis of enantiomerically pure 4-alkylsubstituted tryptophan derivatives by a combination of organometallic reactions with enantioselective enzymatic transformations. Tetrahedron Lett..

[25] Iwao M., Iwao F. (1997). A concise total synthesis of (±)-cis-and (±)-trans-clavicipitic acids by combinational use of directed lithiation and flouride ion-induced elimination-addition reaction of 1-(triisopropylsilyl)gramine derivatives. Tetrahedron.

[26] Shinohar H., Fukuda T., Iwao M. (1999). A formal synthesis of optically active clavicipitic acids, unusual azepinoindole-type ergot alkaloids. Tetrahedron.

[27] Fukuda T., Akashima H., Iwao M. (2005). Synthesis of 3,4,5-trisubstituted indoles via iterative directed lithiation of 1-(triisopropylsilyl)gramines. Tetrahedron.

[28] Huisman M., Rahaman M., Asad S., Oehm S., Novin S., Rheingold A.L., Hossain M.M. (2019). Total synthesis of tryprostatin B: Synthesis and asymmetric phase-transfer-catalyzed reaction of prenylated gramine salt. Org. Lett..

[29] Vlasova M.I., Kogan N.A. (1983). New synthesis of substituted gramines. Chem. Heterocycl. Compd..

[30] Chernikova I.B., Spirikhin L.V., Yunusov M.S. (2017). Synthesis of N-1-skatyl uracil derivatives. Chem. Nat. Compd..

[31] Ke S., Shi L., Cao X., Yang Q., Liang Y., Yang Z. (2012). Heterocycle-functional gramine analogues: Solvent- and catalyst-free synthesis and their inhibition activities against cell proliferation. Eur. J. Med. Chem..

[32] Aydin H., Korkusuz E., Yildirim İ. (2021). Multicomponent reactions of indoles with 1-amino-5-aroyl-4-aryl-1H-pyrimidin-2-ones/-thiones: One-pot three component synthesis of novel gramine analogues. Lett. Org. Chem..

[33] Hogan I., Jenkins P.D., Sainsbury M. (1990). The chemistry of the pyrido[4,3-b]carbazoles part 15 the synthesis of unsymmetrically 1,4-disubstituted carbazoles and their use in the synthesis of 6H-Pyrido-[4,3-b]carbazoles. Tetrahedron.

[34] Cravotto G., Demartin F., Palmisano G., Penoni A., Radice T., Tollari S. (2005). Novel cyclometallated Pd(II) and Pt(II) complexes with indole derivatives and their use as catalysts in Heck reaction. J. Organomet. Chem..

[35] Wang S.-M., Liu C.-C. (2009). Enterovirus 71: Epidemiology, pathogenesis and management. Expert Rev. Anti-Infect..

[36] Li Z.H., Li C.M., Ling P., Shen F.H., Chen S.H., Liu C.C., Yu C.K., Chen S.H. (2008). Ribavirin reduces mortality in enterovirus 71-infected mice by decreasing viral replication. J. Infect. Dis..

[37] Liu M.L., Lee Y.P., Wang Y.F., Lei H.Y., Liu C.C., Wang S.M., Su I.J., Wang J.R., Yeh T.M., Chen S.H. (2005). Type I interferons protect mice against enterovirus 71 infection. J. Gen. Virol..

[38] Wei Y., Shi L., Wang K., Liu M., Yang Q., Yang Z., Ke S. (2014). Discovery of gramine derivatives that inhibit the early stage of EV71 replication in vitro. Molecules.

[39] Lu A., Wang T., Hui H., Wei X., Cui W., Zhou C., Li H., Wang Z., Guo J., Ma D. (2019). Natural products for drug discovery: Discovery of gramines as novel agents against a plant virus. J. Agric. Food Chem..

[40] Ji X.F., Wang Z.W., Dong J., Liu Y.X., Lu A.D., Wang Q.M. (2016). Discovery of topsentin alkaloids and their derivatives as novel antiviral and anti-phytopathogenic fungus agents. J. Agric. Food Chem..

[41] Zhao X., Zhao L., Zhao Y., Huang K., Gong W., Yang Y., Zhao L., Xia X., Li Z., Sheng F. (2021). 3-Indoleacetonitrile is highly effective in treating influenza a virus infection in vitro and in vivo. Viruses.

[42] Yang C., Yu Y., Sun W., Xia C. (2014). Indole derivatives inhibited the formation of bacterial biofilm and modulated Ca^2+^ efflux in diatom. Mar. Pollut. Bull..

[43] Fu R., Lu L., Li Z., Yu Q., Zhang X. (2014). Antibacterial effects of 31 kinds of traditional chinese medicine monomers on MRSA in vitro. Chin. Pharm..

[44] Maver M., Escudero-Martinez C., Abbott J., Morris J., Hedley P.E., Mimmo T., Bulgarelli D. (2021). Applications of the indole-alkaloid gramine modulate the assembly of individual members of the barley rhizosphere microbiota. PeerJ.

[45] Schütz V., Frindte K., Cui J., Zhang P., Hacquard S., Schulze-Lefert P., Knief C., Schulz M., Dörmann P. (2021). Differential impact of plant secondary metabolites on the soil microbiota. Front. Microbiol..

[46] Schreiber K.J., Nasmith C.G., Allard G., Singh J., Subramaniam R., Desveaux D. (2011). Found in translation: High-throughput chemical screening in *Arabidopsis thaliana* identifies small molecules that reduce fusarium head blight disease in wheat. Mol. Plant Microbe Interact..

[47] Wollein U., Bracher F. (2011). The gramine route to pyrido[4,3-b]indol-3-ones-identification of a new cytotoxic lead. Sci. Pharm..

[48] Dan W., Cao Y., Sun Y., Zhang J., Liu J., Gao J., Han R., Dai J. (2023). Novel N^1^ or N^9^ modified α-carboline analogues as potential ligands in Alzheimer’s disease therapy: Synthesis and neurobiological activity evaluation. Bioorg. Chem..

[49] Batista C.R.A., Gomes G.F., Candelario-Jalil E., Fiebich B.L., Oliveira A.C.P. (2019). Lipopolysaccharide-induced neuroinflammation as a bridge to understand neurodegeneration. Int. J. Mol. Sci..

[50] Han R., Yuan T., Yang Z., Zhang Q., Wang W.-W., Lin L.-B., Zhu M.-Q., Gao J.-M. (2021). Ulmoidol, an unusual nortriterpenoid from *Eucommia ulmoides* Oliv. Leaves prevents neuroinflammation by targeting the PU.1 transcriptional signaling pathway. Bioorg. Chem..

[51] Magalhães S.C.Q., Fernandes F., Cabrita A.R.J., Fonseca A.J.M., Valentão P., Andrade P.B. (2017). Alkaloids in the valorization of European *Lupinus* spp. seeds crop. Ind. Crop. Prod..

[52] Xie X., Xiao H., Ding F., Zhong H., Zhu J., Ma N., Mei J. (2014). Over-expression of prolyl hydroxylase-1 blocks NF-κB-mediated cyclin D1 expression and proliferation in lung carcinoma cells. Cancer Genet..

[53] Bernardes V.F., Gleber-Netto F.O., Sousa S.F., Rocha R.M., Aguiar M.C. (2013). EGFR status in oral squamous cell carcinoma: Comparing immunohistochemistry, FISH and CISH detection in a case series study. BMJ Open.

[54] Ramu A., Kathiresan S., Ramadoss H., Nallu A., Kaliyan R., Azamuthu T. (2018). Gramine attenuates EGFR-mediated inflammation and cell proliferation in oral carcinogenesis via regulation of NF-κB and STAT3 signaling. Biomed. Pharmacother..

[55] Lu X., Lu F., Yu J., Xue X., Jiang H., Jiang L., Yang Y. (2021). Gramine promotes functional recovery after spinal cord injury via ameliorating microglia activation. J. Cell. Mol. Med..

[56] Torre L.A., Bray F., Siegel R.L., Ferlay J., Lortet-Tieulent J., Jemal A. (2015). Global cancer statistics, 2012. CA-Cancer J. Clin..

[57] Shklar G. (1999). Development of experimental oral carcinogenesis and its impact on current oral cancer research. J. Dent. Res..

[58] Kumar R.A., Suresh K. (2014). Chemopreventive potential of gramine against 7, 12-dimethylbenz [a] anthracene induced hamster buccal pouch carcinogenesis. Int. J. Mod. Res. Rev..

[59] Ramu A., Kathiresan S., Ahmed B.A. (2017). Gramine inhibits angiogenesis and induces apoptosis via modulation of TGF-beta signalling in 7,12 dimethylbenz[a]anthracene (DMBA) induced hamster buccal pouch carcinoma. Phytomedicine.

[60] Zhang X.-H., Guo Q., Wang H.-Y., Li Y.-H., Khamis M.Y., Ma L.-Y., Wang B., Liu H.-M. (2021). Gramine-based structure optimization to enhance anti-gastric cancer activity. Bioorg. Chem..

[61] Alnaim A.S. (2023). Formulation, characterization, and cytotoxic effect of PVA incorporated iron oxide nanoparticles of gramine using HCT-116 cell line in vitro. Indian J. Pharm. Educ. Res..

[62] Cousins K.A.Q., Bove J., Giannini L.A.A., Kinney N.G., Balgenorth Y.R., Rascovsky K., Lee E.B., Trojanowski J.Q., Grossman M., Irwin D.J. (2021). Longitudinal naming and repetition relates to AD pathology and burden in autopsy-confirmed primary progressive aphasia. Alzh. Dement-TRCI.

[63] Iqbal K., Liu F., Gong C.X. (2016). Tau and neurodegenerative disease: The story so far. Nat. Rev. Neurol..

[64] Zhang M., Yogesha S.D., Mayfield J.E., Gill G.N., Zhang Y. (2013). Viewing serine/threonine protein phosphatases through the eyes of drug designers. FEBS J..

[65] Anand R., Gill K.D., Mahdi A.A. (2014). Therapeutics of Alzheimer’s disease: Past, present and future. Neuropharmacology.

[66] Lajarin-Cuesta R., Nanclares C., Arranz-Tagarro J.A., Gonzalez-Lafuente L., Arribas R.L., Brito M.A., Gandia L., Rios C.I. (2016). Gramine derivatives targeting Ca^2+^ channels and ser/thr phosphatases: A new dual strategy for the treatment of neurodegenerative diseases. J. Med. Chem..

[67] Gonzalez D., Arribas R.L., Viejo L., Lajarin-Cuesta R., Rios C.L. (2018). Substituent effect of N-benzylated gramine derivatives that prevent the PP2A inhibition and dissipate the neuronal Ca^2+^ overload, as a multitarget strategy for the treatment of Alzheimer’s disease. Bioorgan. Med. Chem..

[68] Lajarín-Cuesta R., Arribas R.L., Nanclares C., García-Frutos E.M., Gandía L., Ríos C.L. (2018). Design and synthesis of multipotent 3-aminomethylindoles and 7-azaindoles with enhanced protein phosphatase 2A-activating profile and neuroprotection. Eur. J. Med. Chem..

[69] Jadhav G.B., Sable R.R. (2023). Neuroprotective impact of zingerone and gramine on scopolamine-induced amnesia model. J. Pharm. Negat. Result..

[70] Spitzer N., Edwards D.H., Baro D.J. (2008). Conservation of structure, signaling and pharmacology between two serotonin receptor subtypes from decapod crustaceans, *Panulirus interruptus* and *Procambarus clarkia*. J. Exp. Biol..

[71] Adkins E.M., Barker E.L., Blakely R.D. (2001). Interactions of tryptamine derivatives with serotonin transporter species variants implicate transmembrane domain I in substrate recognition. Mol. Pharmacol..

[72] Yin X.-J., Huang X.-Y., Ma Y.-B., Geng C.-A., Li T.-Z., Chen X.-L., Yang T.-H., Zhou J., Zhang X.-M., Chen J.-J. (2017). Bioactivity-guided synthesis of gramine derivatives as new MT1 and 5-HT1A receptors agonists. J. Asian Nat. Prod. Res..

[73] Katz P.S., Frost W.N. (1995). Intrinsic neuromodulation in the Tritonia swim CPG: Serotonin mediates both neuromodulation and neurotransmission by the dorsal swim interneurons. J. Neurophysiol..

[74] Couper J.M., Leise E.M. (1996). Serotonin injections induce metamorphosis in larvae of the gastropod mollusc *Ilyanassa obsolete*. Biol. Bull..

[75] Litosch I., Fradin M., Kasaian M., Lee H.S., Fain J.N. (1982). Regulation of adenylate cyclase and cyclic AMP phosphodiesterase by 5-hydroxytryptamine and calcium ions in blowfly salivary-gland homogenates. Biochem. J..

[76] Renaud F., Parisi E., Capasso A., Prisco P. (1983). On the role of serotonin and 5-methoxy-tryptamine in the regulation of cell division in sea urchin eggs. Dev. Biol..

[77] Niacaris T., Avery L. (2003). Serotonin regulates repolarization of the *C. elegans* pharyngeal muscle. J. Exp. Biol..

[78] Froldi G., Silvestrin B., Dorigo P., Caparrotta L. (2004). Gramine: A vasorelaxing alkaloid acting on 5-HT2A receptors. Planta Med..

[79] Sheng W.L., Chen W.Y., Yang X.L., Zhong Y.M., Weng S.J. (2015). Co-expression of two subtypes of melatonin receptor on rat M1-type intrinsically photosensitive retinal ganglion cells. PLoS ONE.

[80] Orchard I., Lange A.B. (1986). Pharmacological profile of octopamine receptors on the lateral oviducts of the locust, *Locusta migratoria*. J. Insect Physiol..

[81] Yang J., Kong X.-D., Zhu-Salzman K., Qin Q.-M., Cai Q.N. (2021). The key glutathione S-transferase family genes involved in the detoxification of rice gramine in brown planthopper *Nilaparvata lugens*. Insects.

[82] Griffiths M.R., Strobel B.W., Hama J.R., Cedergreen N. (2021). Toxicity and risk of plant-produced alkaloids to *Daphnia magna*. Environ. Sci. Eur..

[83] Zohdi E., Abbaspour M. (2019). Harmful algal blooms (red tide): A review of causes, impacts and approaches to monitoring and prediction. Int. J. Environ. Sci. Technol..

[84] Mary M.A., Rashel R.H., Patiño R. (2021). Growth inhibition of the harmful alga *Prymnesium parvum* by plant-derived products and identification of ellipticine as highly potent allelochemical. J. Appl. Phycol..

[85] Xu L., Su Y., Yang X., Bai X., Wang Y., Zhuo C., Meng Z. (2023). Gramine protects against pressure overload-induced pathological cardiac hypertrophy through Runx1-TGFBR1 signaling. Phytomedicine.

[86] Kozanecka-Okupnik W., Sierakowska A., Kowalczyk I., Mrówczyńska L., Jasiewicz B. (2022). New triazole-bearing gramine derivatives-synthesis, structural analysis and protective effect against oxidative haemolysis. Nat. Prod. Res..

